# Understanding the Relationship between Atherogenic Index of Plasma and Cardiovascular Disease Risk Factors among Staff of an University in Malaysia

**DOI:** 10.1155/2018/7027624

**Published:** 2018-07-04

**Authors:** Myat Su Bo, Whye Lian Cheah, Soe Lwin, Tin Moe Nwe, Than Than Win, Myint Aung

**Affiliations:** ^1^Department of Basic Medical Sciences, Faculty of Medicine and Health Sciences, Universiti Malaysia Sarawak, 94300 Kota Samarahan, Sarawak, Malaysia; ^2^Department of Community Medicine and Public Health, Faculty of Medicine and Health Sciences, Universiti Malaysia Sarawak, 94300 Kota Samarahan, Sarawak, Malaysia; ^3^Department of Obstetrics and Gynecology, Faculty of Medicine and Health Sciences, Universiti Malaysia Sarawak, 94300 Kota Samarahan, Sarawak, Malaysia; ^4^Department of Medicine, Faculty of Medicine and Health Sciences, Universiti Malaysia Sarawak, 94300 Kota Samarahan, Sarawak, Malaysia

## Abstract

**Background:**

Atherogenic index of plasma (AIP) was found to be one of the strongest markers in predicting the cardiovascular disease (CVD) risk. This study was to determine the AIP and its relationship with other CVD risk factors.

**Materials and Methods:**

This cross-sectional study was done among 349 staff of a public university in Sarawak. Data were collected using questionnaire, blood sampling, and anthropometric and blood pressure measurement. Data were analyzed using IBM SPSS version 20.

**Results:**

A total of 349 respondents participated with majority females (66.8%), aged 38.5 ± 7.82 years. Nearly 80% of the respondents were overweight and obese, 87.1% with high and very high body fat, and 46.9% with abnormal visceral fat. For AIP category, 8.9% were found to be in intermediate and 16.4% were at high risk. Elevated lipid profile showed that total cholesterol (TC) is 15.5%, low density lipoprotein (LDL) is 16.1%, and triglyceride (TG) is 10.6%. AIP was significantly correlated with body mass index (*r*=0.25), visceral fat (*r*=0.37), TC (*r*=0.22), LDL (0.24), HDL (*r*=−0.72), TG (*r*=0.84), glucose (*r*=0.32), systolic blood pressure (*r*=0.22), and diastolic blood pressure (*r*=0.28).

**Conclusion:**

It indicated that AIP is associated with other CVD risk factors. Modification of lifestyle is strongly recommended.

## 1. Introduction

Noncommunicable diseases (NCDs) are the major health problems in the world today. Majority of premature deaths are due to these diseases, and most of them can be prevented by practicing healthy lifestyle and undergoing early intervention programs. According to the WHO Global Action Plan for the prevention and control of NCDs (2013–2020), it targeted that 25% reduction in overall mortality from NCDs including cardiovascular disease, cancer, diabetes mellitus, and chronic respiratory diseases is achieved [1].

In Malaysia, due to rapid urbanization and industrialization, NCDs become the obvious cause of mortality and morbidity because it contributes to an estimated 73% of total reported deaths. Among them, the most common cause is cardiovascular disease. An estimated 35% of deaths occur in individuals aged less than 60 years, which are mainly in working age group. A report from National Health and Morbidity Survey (2015) identified that at least 63% of adults aged 18 years and above had at least one NCD risk factor [2].

Understanding modifiable risk factors for CVD such as smoking, hypertension, diabetes, overweight, and high cholesterol can help to prevent and reduce disease burden. Dyslipidemia is defined as elevated plasma concentration of lipids (triglyceride and total cholesterol) and their related blood-transporting lipoproteins: HDL cholesterol, LDL cholesterol, and VLDL cholesterol [3–6]. In the absence of an unfavorable lipid profile, the possible consequence of CVD cannot be ruled out [7].

The atherogenic index of plasma (AIP) is a critical index that can be used as a stand-alone index for cardiac risk estimation [8]. Changes in the levels of any lipid profile make the individuals more prone to get the chance of atherosclerotic complications [9]. It is defined as logarithm [log] of the ratio of plasma concentration of TG to HDL-C and is strongly correlated with CVD risks. It can act as an adjunct over the individual lipid profile. AIP is the best determinant for fractionated esterification rate of HDL-C and more useful than routine lipid parameters [10, 11]. It can be used as a diagnostic indicator when the other atherogenic risk parameters appear normal [12]. The AIP calculation estimates the values of “zone of atherogenic risk” [13].

Improved quality of life has brought upon an increase in the average life expectancy from 68.9 to 73.5 years for males and from 71.7 to 74.5 years for females between 1990 and 2007 [14]. However, at least one type of CVD risk factor is still present in 61% of the adult population [15]. Work-related stress also can increase the risk relating to cardiovascular disease. Some people when they get stressed, they tend to eat more, and most of the time they do not realize the proportion and type of food that they consumed, and it eventually leads to obesity [16]. Health plays a significant role in meeting the job demand of government workers. For instance, in the universities, the staff need to perform well as to ensure the quality of the students of their respective departments and to bring up a good reputation of the department they work in. It is vital for government workers to maintain a healthy body by being aware of their health status so as to be able to perform optimally in their jobs [17].

This study implemented the relationship between AIP and other CVDs risks among university staff in order to highlight that AIP rather than lipid profile is the predictor for CVDs. By performing this study, we can detect the early cases among healthy staff and provide advices such as lifestyle changes, increased physical activity, and healthy diet.

## 2. Materials and Methods

This cross-sectional study was carried out among staff of Universiti Malaysia Sarawak. All respondents who fulfilled the inclusion criteria of no current acute illness and no known history of diabetes mellitus, hypertension, heart disease, liver disease, and renal disease were selected. Those who were taking lipid-lowering drugs were excluded from the study. The study was conducted from October 2016 to April 2017. The study was approved by the Medical Ethical Committee of Universiti Malaysia Sarawak [UNIMAS/NC-21.02/03-02 Jld.2 (22)]. All respondents were briefed, and they signed informed consent.

Sample size was calculated using EpiInfo (version 3), based on the sampling frame of 2271, prevalence of overweight and obesity which is 53.1% [18], confidence level of 95%, attrition rate of 10%, and estimated minimum sample size needed is 370. All the eligible respondents in all the administrative divisions and faculties who met the inclusion criteria were invited to participate in the study. Blood samples were collected from all respondents after 10–12 hours fasting. A private laboratory was engaged in assisting in the blood collection and carry out the respective test. According to the Malaysian clinical practice guidelines on management of dyslipidemia [19], hypercholesterolemia is classified as total cholesterol of more than 6.3 mmol/L, high LDL cholesterol is defined as more than 4.1 mmol/L, low HDL cholesterol is classified as less than 1 mmol/L for men and less than 1.3 mmol/L for women, and high triglyceride is defined as more than 2.3 mmol/L.

Atherogenic index was calculated by using the following formula: log_10_ (TG/HDL-C) [6]. It can be classified according to the values obtained: −0.3 to 0.1 for low risk, 0.1 to 0.24 for medium, and more than 0.24 for high risk of CVD [20]. For the fasting blood glucose, it can be classified as following: values <6.1 mmol/L are normal, and values ≥6.1 mmol/L are high [21]. Height was measured by using a stadiometer (model: SECA 213, Germany). The participants were asked to stand upright barefooted on a flat surface with their back of the heels and occiput against the body meter. The body must be in straight line and required to look straight ahead [22]. The weight, BMI, percentage of body fat, and visceral fat were measured through bioelectrical impedance analysis (model: HBF-375, Omron Healthcare Co., Ltd., Kyoto, Japan). For the measurement of body composition, respondents were asked to stand barefooted with knees and back straight and look straight ahead. The arms were horizontally raised and extended to a 90° angle to their bodies, and the elbows were extended straight. The palms should firmly grip the electrodes. The readings were displayed on the handheld meter of the equipment.

Body mass index is classified as follows: underweight (BMI < 18.5 kg/m^2^), normal (18.5 to 22.9 kg/m^2^), overweight (BMI ≥ 23 kg/m^2^), and obese (BMI ≥ 27.5 kg/m^2^) [23]. The percentage of body fat can be classified as follows: for men <10% as low, 10 to 19.9% as normal, 20 to 24.9% as high, and ≥25% as very high, and for the women, <20% as low, 20 to 29.9% as normal, 30 to 34.9% as high, and ≥35% as very high body fat [24]. For the visceral fat, it can be classified as follows: normal (1 to 9%), high (10 to 14%), and very high (15 to 30%) for both genders [25].

Blood pressure was measured by using a digital blood pressure monitor (model: HBP-1300, Omron Healthcare Co., Ltd., Kyoto, Japan) in the sitting position with the hand resting on the examining table, and the cubital fossa was supported at the level of the heart. The blood pressure was measured after the respondents had rested for at least 5 minutes. Two measurements were taken for each participant at 15 minutes apart [26]. All monitors were calibrated with the manual sphygmomanometer prior to each data collection. According to the Malaysian clinical practice guidelines on management of hypertension (2013), prehypertension is classified as systolic blood pressure 120 to 139 mmHg or diastolic blood pressure 80 to 89 mmHg, and high blood pressure is classified as systolic blood pressure ≥ 140 mmHg or diastolic blood pressure ≥ 90 mmHg [27].

Data entry and data analysis were performed by using IBM Statistical Package for Social Science Program (SPSS) version 20. Descriptive and inferential statistics were carried out based on 95% confidence interval with *p* value less than 0.05 as significant. Descriptive statistics were brief descriptive coefficients that summarize the given data set while inferential statistics made reference about populations using data drawn from the populations.

## 3. Results

A total of 349 respondents participated in the study. The study population consisted of 66.8% females with mean age of 38.5 year ±7.82. More than 70% of them were Malay, and the majority of participants (64.8%) obtained their tertiary educational level. The higher percentage of the employment group was administrative staff (80.5%). As for the marital status, 74.8% of them were married. The details of the respondents are presented in Table 1.

About 80% of the respondents were overweight and obese, with mean BMI of 27.2 ± 5.60 kg/m^2^. More than 80% of the respondents were found to have high and very high body fat percentage, and 46.9% had high and very high visceral fat percentage. More than 30% of the respondents had prehypertension, and 20.3% had hypertension. For the fasting blood glucose, 12% had elevated blood glucose level (≥6.1 mmol/L). About 15% of the respondents had high total cholesterol, 16.1% had high LDL cholesterol, and about 10% had high triglyceride. In terms of HDL cholesterol, 16% of the respondents reported to be in the low level. About 16% of the respondents had high AIP. Details on the cardiovascular risk factors of the respondents and their association with gender are presented in Tables 2 and 3.

For comparison between male and female respondents, significant differences were observed for SBP, DBP, LDL-C, HDL-C, TG, AIP, body fat percentage, and visceral fat with *p* < 0.01. Other indicators such as BMI, blood glucose, and total cholesterol were found to be insignificant between males and females. Except for HDL-C and body fat percentage, all other significant indicators showed higher findings in males as compared to females. The details of the finding are presented in Table 3.

AIP had significantly positive correlation with BMI (*r*=0.246), visceral fat (*r*=0.370), total cholesterol (*r*=0.218), LDL cholesterol (*r*=0.237), triglyceride (*r*=0.839), glucose (*r*=0.324), systolic blood pressure (*r*=0.217), and diastolic blood pressure (*r*=0.283) (*p* < 0.001). There was a negative correlation between AIP and HDL cholesterol (*r*=−0.719). The details of the finding are presented in Table 4.

## 4. Discussion

In worldwide, the prevalence of overweight and obesity in adults aged 18 years and above was 39% and 13%, respectively, in 2016 [28]. In Malaysia, the report from the National Health and Morbidity Survey (2015) stated that the prevalence of overweight and obesity was 33.4% and 30.6%, respectively. In this study, a majority of the participants were overweight and obese (79.9%), and it indicated that an alarming sign of higher prevalence of obesity. It was consistent with the previous studies [29, 30] where more than half of their civil servants screened were obese. It indicated rapid urbanization and adaptation of more sedentary lifestyle leading to unhealthy outcomes.

This study also reported that more than 80% of the participants had high and very high body fat percentage, consistent with the study done by Chai and Chee [31]. In assessing adults' nutritional status, the literature had elucidated that the use of body fat percentage is a more accurate parameter in predicting CVD risk as BMI does not differentiate between body fat and lean mass [32, 33]. Between genders, female respondents were found to have higher prevalence of over fat (35.2%) compared to males (26.7%). It would be reasonable that women are more effective in subcutaneous fat storage than men. But for the men, intraabdominal fat including visceral adipose fat is more preferable. Compared to men, women have relatively more adipose tissue in the hip and thigh [34]. Therefore, in this study, even though BMI of the male was higher than female, the body fat percentage of female was higher than male.

Individual with more visceral adipose tissue has a higher chance to get CVD complications. The reason for that is visceral adipose tissue, which is mainly drained by the portal venous system and subsequently drained into the liver, in which it causes insulin resistance [35]. When compared to subcutaneous fat, the free fatty acid (FFA) mobilization is more rapid in visceral fat and subsequently increases the FFA levels in the systemic circulation [36]. Besides that, visceral fat has a stronger lipolytic effect from catecholamine and weaker antilipolytic effect from insulin hormone which may probably be due to decrease in insulin receptor affinity in that tissue [37]. These excess free fatty acids may cause the enhancement of lipid synthesis and gluconeogenesis, as well as insulin resistance, resulting in hyperlipidemia, glucose intolerance, hypertension, and finally atherosclerosis [35]. In this study, the prevalence of high visceral fat was 46.8%, which comprised almost half of the participants. Chai and Chee [31] in their study reported that 4 out of 15 subjects manifested with high visceral fat accumulation. Consistent with the literature, the finding reported that visceral fat percentage of men was higher (12.7%) than women (9.1%), similar to the results of the study conducted by Ajani et al. [30].

Visceral fat produced a substance like tumor necrosis factor-*α* (TNF-*α*) that induces insulin resistance which played an important role in elevation of blood pressure by means of vasoactive adepocytokines (e.g., angiotensinogen) [38, 39]. In this study, more than 30% of participants had prehypertension and 20.1% had hypertension. In Malaysia, the prevalence of hypertension in the age 30 years and above was 42.0% [40].

About 12% of participants in this study had increased fasting blood glucose levels, which was slightly lower than the findings from NHMS (2015), in which it is indicated that the overall prevalence was 17.5% [2]. Therefore, hyperglycemia, abnormal lipid profile, and to some extent hypertension were all involved in development of atherosclerosis [20, 41, 42].

During the last decades, the epidemiologists and clinicians evaluated that the risks of CVDs were mainly based on the fact that LDL-C was not optimal, especially in those with intermediate risks. Many studies reported the importance of many lipid ratios or atherogenic indexes. These indexes were strong indicators of the CVD risk by its expressions of imbalance between atherogenic and antiatherogenic lipoproteins [43, 44]. Atherogenic index of plasma (AIP) has emerged as a predictive marker for plasma atherogenicity [45]. It was associated with HDL, LDL, and VLDL particle sizes and predicted the CVD risk [10]. AIP was the most sensitive marker compared with other three atherogenic indices like Castelli's risk index-I (TC/HDL-C), Castelli's risk index-II (LDL-C/HDL-C), and atherogenic coefficient (TC-HDL-C/HDL-C) [7, 46]. Isolated elevation in triglyceride increases the CHD risk but these effects can be balanced by cardioprotective lipoprotein of HDL cholesterol [47]. Moreover, if the other atherogenic risk parameters appear normal, AIP may be the diagnostic alternative [12].

In this study, 8.9% of participants had intermediate risk and 16.4% had a high risk of AIP. Furthermore, there was a significant positive correlation between AIP and total cholesterol, LDL cholesterol, and triglyceride and significant negative correlation between AIP and HDL cholesterol. It was supported by the findings of the previous study which reported that significant increase in AIP was detected with increasing total cholesterol, triglyceride, and LDL cholesterol and decreasing HDL cholesterol [48]. Moreover, among lipid profiles, AIP was positively higher correlated with triglyceride (*r*=0.839) and negatively higher correlated with HDL cholesterol (*r*=−0.719). Therefore, AIP is the strongest marker in the risk assessment of CVD instead of the other indexes.

Even though in this study, there were 8.9% and 16.4% for intermediate risk and high risk of AIP, respectively, the mean age was 38.5 ± 7.82. There is a need for this age group to follow-up for especially those who had high and very high body fat percentage, since there was a positive correlation between AIP and BMI, visceral fat, glucose, systolic, and diastolic blood pressure in this study.

## 5. Limitations

This study was carried out among the staff of the studied university and cannot be generalized for general population. Furthermore, it was a cross-sectional study; therefore, no causal relationship can be established. The findings of this study are also limited by the absence of physical activity and dietary assessment. Thus, interpretation of data must be done cautiously. Future studies could overcome these limitations by incorporating physical activity, dietary intake pattern, and the influences of social and environmental factors.

## 6. Conclusion

This study showed that there was a significant correlation between AIP and CVD risk factors (BMI, visceral fat, body fat, total cholesterol, LDL cholesterol, triglyceride, glucose, and HDL cholesterol) among the studied samples. Based on these findings, for prevention of the CVD risk and early intervention programs such as exercise, dietary control, and monitoring of AIP especially for those who are in the high risk category should be carried out regularly.

## Figures and Tables

**Table 1:** Sociodemographic characteristics of the participants (*N*=349).

	*n* (%)	Mean ± SD
Age (years)		38.5 ± 7.82

Gender		
Male	116 (33.2)	
Female	233 (66.8)	

Ethnic groups		
Malay	252 (72.2)	
Chinese	12 (3.4)	
Others (including Iban and Bidayuh)	85 (24.4)	

Educational levels		
Primary	8 (2.3)	
Secondary	115 (33.0)	
Tertiary	226 (64.8)	

Employment positions		
Administrative	281 (80.5)	
Lecturer	68 (19.5)	

Marital status		
Single (including widow and divorcee)	88 (25.2)	
Married	261 (74.8)	

**Table 2:** Cardiovascular disease risk factors of the participants (*N*=349).

	*n* (%)	Mean ± SD
BMI (kg/m^2^)		27.2 ± 5.60
Normal	70 (20.1)	
Overweight and obese	279 (79.9)	

Body fat (%)		32.4 ± 6.51
Normal	45 (12.9)	
High and very high	304 (87.1)	

Visceral fat (%)		10.2 ± 5.42
Normal	185 (53.1)	
High and very high	164 (46.9)	

Blood pressure		
SBP (mmHg)		127.3 ± 18.39
DBP (mmHg)		77.8 ± 12.43
Normal	150 (43.0)	
Prehypertension	128 (36.7)	
Hypertension	71 (20.3)	

Blood glucose (mmol/L)		5.3 ± 1.3
Normal	307 (88)	
High	42 (12)	

Total cholesterol (mmol/L) (*n*=348)		5.4 ± 1.06
Optimal	157 (45.1)	
Borderline	137 (39.4)	
High	54 (15.5)	

LDL-C (mmol/L) (*n*=342)		3.3 ± 0.96
Optimal	70 (20.5)	
Borderline	217 (63.5)	
High	55 (16.1)	

HDL-C (mmol/L)		1.4 ± 0.37
Low	56 (16.0)	
Average	159 (45.6)	
Optimal	134 (38.4)	

TG (mmol/L)		1.4 ± 1.11
Optimal	259 (74.2)	
Borderline	53 (15.2)	
High	37 (10.6)	

AIP (*n*=347)		−0.1 ± 0.30
Low risk	259 (74.6)	
Intermediate risk	31 (8.9)	
High risk	57 (16.4)	

BMI: body mass index; SBP: systolic blood pressure; DBP: diastolic blood pressure; LDL-C: low-density lipoprotein cholesterol; HDL-C: high-density lipoprotein cholesterol; TG: triglyceride; AIP: atherogenic index of plasma.

**Table 3:** Cardiovascular disease risk factors between male and female (*n*=349).

	Male	Female	*p* value
	Mean (±SD)	Mean (±SD)	
BMI (kg/m^2^)	27.8 (6.81)	26.9 (4.88)	0.140
Body fat (%)	26.7 (6.25)	35.2 (4.48)	<0.0001^*∗∗*^
Visceral fat (%)	12.7 (4.99)	9.1 (5.23)	<0.0001^*∗∗*^
SBP (mmHg)	133.3 (16.02)	124.3 (18.78)	<0.0001^*∗∗*^
DBP (mmHg)	81.8 (12.27)	75.9 (12.06)	<0.0001^*∗∗*^
Blood glucose (mmol/L)	5.3 (1.32)	5.3 (1.30)	0.838
Total cholesterol (mmol/L)	5.5 (0.98)	5.3 (1.09)	0.107
LDL-C (mmol/L)	3.5 (0.92)	3.2 (0.97)	<0.0001^*∗∗*^
HDL-C (mmol/L)	1.3 (0.22)	1.5 (0.40)	0.007^*∗*^
TG (mmol/L)	1.8 (1.41)	1.3 (0.88)	<0.0001^*∗∗*^
AIP	0.07 (0.30)	−0.13 (0.28)	<0.0001^*∗∗*^

BMI: body mass index; SBP: systolic blood pressure; DBP: diastolic blood pressure; LDL-C: low-density lipoprotein cholesterol; HDL-C: high density lipoprotein cholesterol; TG: triglyceride; AIP: atherogenic index of plasma; ^*∗*^significant at *p* < 0.05; ^*∗∗*^significant at *p* < 0.001.

**Table 4:** Correlation between AIP and other CVD risk factors.

AIP	*r*	*p* value
BMI	0.246	<0.001
VF	0.237	<0.001
BF	0.015	—
TC	0.218	<0.001
LDL-C	0.237	<0.001
HDL-C	−0.719	<0.001
TG	0.839	<0.001
Glucose	0.324	<0.001
SBP	0.217	<0.001
DBP	0.283	<0.001

AIP: atherogenic index of plasma; BMI: body mass index; VF: visceral fat; BF: body fat; TC: total cholesterol; LDL-C: low-density lipoprotein cholesterol; HDL-C: high-density lipoprotein cholesterol; TG: triglyceride; SBP: systolic blood pressure; DBP: diastolic blood pressure.

## Data Availability

The data used to support the findings of this study are available from the corresponding author upon request.
